# Prevalence of prior HIV testing and associated factors among MSM in Zhejiang Province, China: a cross-sectional study

**DOI:** 10.1186/s12889-016-3806-2

**Published:** 2016-11-10

**Authors:** Runhua Li, Xiaohong Pan, Qiaoqin Ma, Hui Wang, Lin He, Tingting Jiang, Dayong Wang, Yan Zhang, Xingliang Zhang, Shichang Xia

**Affiliations:** 1 Zhejiang Provincial Center for Disease Control and Prevention, No.3399, Binsheng Road, Hangzhou, Zhejiang Province 310051 People’s Republic of China; 2 Department of Epidemiology and Health Statistics, School of Medicine, Ningbo University, Ningbo, Zhejiang Province People’s Republic of China; 3 Wenzhou Center for Disease Control and Prevention, Wenzhou, Zhejiang Province People’s Republic of China; 4 Ningbo Center for Disease Control and Prevention, Ningbo, Zhejiang Province People’s Republic of China; 5 Hangzhou Center for Disease Control and Prevention, Hangzhou, Zhejiang Province People’s Republic of China

**Keywords:** Men who have sex with men (MSM), HIV testing, Associated factors, Respondent-driven sampling

## Abstract

**Background:**

Men who have sex with men (MSM) have become one of high-risk population for human immunodeficiency virus (HIV) infection, due to their multiple sex partners and unprotected anal intercourse. Promoting HIV testing is an effective strategy for the prevention and control of HIV infection. We assessed the factors associated with a prior HIV testing history, which could provide guidance for implementation of future HIV intervention programs.

**Methods:**

A cross-sectional study was conducted in three cities of Zhejiang Province, namely, Hangzhou, Ningbo and Wenzhou, using respondent-driven sampling, between December 2013 and June 2014. A face-to-face questionnaire survey was employed to collect relevant information about HIV testing. Univariate and multivariate logistic regression analyses were used to identify the factors associated with a prior HIV testing history.

**Results:**

The adjusted rate of prior HIV testing among MSM in Zhejiang Province was 55.9 %. The adjusted rates of HIV and syphilis infections among MSM in Zhejiang Province were 14.0 % and 11.4 %, respectively. A weighted multivariate analysis showed that MSM of older age were more likely to be tested, as were MSM with higher level of education, self-reported homosexuality and a higher frequency of receiving AIDS/sexually transmitted infections educational intervention in the past year. MSM with suicidal inclination and self-perceived lower possibility of HIV infection were less likely to report ever having undergone an HIV test.

**Conclusions:**

The prevalence of prior HIV testing among MSM in Zhejiang Province, China is low. Effective and more frequent interventional measures should be adopted to improve risk awareness and psychosocial support for younger, less educated MSM, and to encourage more MSM to undergo HIV testing.

## Background

The Sixth National Census showed that in China there are 480 million men aged 15–59, who are sexually active [1], of which 2–5 % have had sex at least once in their lives, with another male. Based on this report, it is estimated that there are 9.6–24 million men who have sex with men (MSM) in China [2]. MSM have become a large high-risk population for human immunodeficiency virus (HIV) infection due to their multiple sex partners and unprotected anal intercourse (UAI). According to the sentinel surveillance data in 2013, the HIV infection rate among MSM is up to 7.3 % all over the country [3]. The proportion of annually reported HIV cases through homosexual transmission has increased from 2.5 % in 2005 to 25.8 % in 2014 [4, 5]. A meta-analysis has shown that MSM are 19.3 times as likely to contract HIV as the general population [6]. The proportion of MSM who are aware of their HIV status is 56.0 % in the US and 73.7 % in Europe [7], while in China only 26 % of MSM know their HIV status [8].

By regular HIV testing and counselling, infected individuals can be identified as early as possible and early detection of HIV status enables patients to avoid risk behaviour through care and support services. Early detection also helps patients to receive timely antiretroviral treatment to reduce HIV viral load in the body, leading to an effective reduction in the spread of HIV. Promoting HIV testing is thus one of effective strategies for HIV prevention and control. Sexually active MSM are recommended to undergo an HIV test every 3–6 months in the US [9]. The Chinese government has established voluntary counselling and testing (VCT) clinics that offer free counselling and testing all over the country. In China, the prevalence of VCT among MSM during their life varies only from 18 to 47.9 % [10–12]. In 2007, the Chinese government sponsored a campaign for HIV/AIDS Prevention and Control among MSM in China for the period between 2007–2010, highlighting cross-sectorial collaboration for HIV prevention, enhanced health education, implementation of the Four Frees and One Care policy, and increased HIV testing. A meta-analysis revealed that the percentage of MSM who reported ever having been tested for HIV has increased to 47 % from 24 %, after the adoption of this plan [13]. However, there is still a great disparity in the prevalence of HIV testing between cities in China and those in other developed countries. For example, the percentage of MSM who reported ever having been tested for HIV is 80.5 % in Australia [14] and 90 % in 21 cities in the USA [15].

Although several studies have identified factors related to the proportion of MSM who reported ever having an HIV test in developed countries, the factors associated with prior HIV testing in China have not been thoroughly assessed. In the US, factors associated with prior HIV testing include older age, having multiple sexual partner during lifetime, having had anal sex with men, having a history of sexually transmitted infections (STIs), and having homosexual orientation [16, 17]. A few studies have assessed the prevalence and associated factors of prior HIV testing in China, by approximate probability sampling such as the Respondent-Driven sampling (RDS) method. A study by Zhang et al. [18] in Chongqing Municipality, China indicated that factors related to prior HIV testing include having a higher education level (bachelor’s degree or higher), a preference for receptive anal sex, and condom use with recent male partners.

Zhejiang Province is located on the southeast coast of the Yangtze River Delta and is one of the most economically developed provinces. The province constitutes the Yangtze River Delta urban agglomeration together with Jiangsu Province, Anhui Province, and Shanghai Municipality in China, which is one of the six biggest world-class international urban agglomerations. There are very few studies about prior HIV testing and its associated factors in these regions. According to the HIV sentinel surveillance data from 2010 to 2013 in Zhejiang province, an HIV antibody positive testing rate has been maintained at around 8 %, among MSM [19]. While many studies have reported HIV testing rate and associated factors in Western countries, in Zhejiang Province, China, very less data is available about these aspects, and therefore the exploration of factors associated with HIV testing in Zhejiang is very important for HIV prevention in this region. In order to acquire data pertaining to the rate of prior HIV testing and its associated factors in Zhejiang Province, we conducted a cross-sectional survey in the three biggest cities of Zhejiang Province, utilizing the RDS method. In our study, we examined the socio-demographic characteristics, risky behaviour variables, and socio-psychological factors, with the goal of identifying factors related to prior HIV testing among MSM in Zhejiang Province. The results of our study can be used to provide guidance for the implementation of HIV intervention programs in the future.

## Methods

### Study population

This cross-sectional study was conducted in three cities of Zhejiang Province, namely, Hangzhou, Ningbo, and Wenzhou, between December 2013 and June 2014. Inclusion criteria among the MSM population in this study were as follows: (1) MSM living in the current city for not less than 3 months when investigated; (2) MSM aged 14 years or older; and (3) MSM who have had oral or anal sex with men, in the last 12 months before this survey. If a respondent in the study met any one of the following criteria, the subject was excluded from this study: (1) was impaired so that cannot clearly understand and answer the questions from the investigation questionnaire due to drunkenness, poisoning or other causes, or had a mental illness or mental retardation, did not fully understand the process of informed consent or did not give informed consent; (2) did not agree to accept the questionnaire survey and/or serological survey; (3) took part in the investigation of subjects without an effective recruiting card; (4) refused to attend the research because of any other reason. The study procedure and sampling strategies are described in detail elsewhere [20]. Briefly, the participants in the study were recruited using RDS. “Seed” candidates were chosen based on the research inclusion and exclusion criteria. The choice of seeds and their ability to develop and drive respondents is directly related to the speed of the balance, which decides the success or failure of the investigation. We recruited MSM as seeds from baths, bars, QQ groups, KTV, clubs, and other different types of venues in accordance with the inclusion criteria. In this study, we consequently recruited eight seeds in Hangzhou, twelve in Ningbo, and five in Wenzhou. Next, the seeds recruited eligible participants, until equilibrium was reached.

### Data collection

A face-to-face questionnaire survey was conducted, including questions about socio-demographic factors, sexual behaviour, sexual partner’s network, social psychological characteristics, history of counselling and testing for HIV, the intention to have an HIV test, the cognition of antiretroviral treatment, and the intention to have early treatment. After the interview, all the participants were tested for HIV and syphilis, free of charge.

Every participant also received a souvenir, as an incentive for participating in this survey, and 100 yuan RMB (approximately 16 US$) or a telephone card for the value of 100 yuan RMB, as a subsidy for phone bills incurred by participants while recruiting three respondents for the study. These incentives were given regardless of whether participants could recruit three eligible partners in a month.

### Ethical considerations

The study was approved by the ethical review board of Zhejiang Provincial Center for Disease Control and Prevention. The research presented no harm or risk to the participants, and their confidentiality and anonymity was strictly preserved. The study was carried out with the intention of improving the health of MSM in Zhejiang Province. All participants gave written informed consent during the survey.

### Statistical analysis

For statistical analysis, the data were entered into EpiData 3.0 via double entry. After cleaning up and verification, they were processed using RDSAT5.6 and SPSS 18.0 software to calculate crude point estimates, population adjustment point estimates and 95 % confidence intervals (95 % CI). Individualized weights were created in SPSS 18.0 using the RDSAT5.6 estimator based on personal social network sizes and patters of recruitment. For univariate and multivariate logistic regression analysis, SPSS18.0 software and individual weights for the outcome variables of prior HIV testing were used. When *P* values were less than 0.05, the variables were subjected to univariate analysis and multivariate logistic regression analysis.

## Results

### Characteristics of participants

A total of 1316 participants were recruited into the study, consisting of 511 participants from Hangzhou, 351 participants from Ningbo, and 454 participants from Wenzhou. All the MSM provided information about prior HIV testing. Their age ranged from 16 to 76 years, with a median of 31 years (interquartile range [IQR], 25–39). The proportion of never-been-married MSM was 64.6 %. About half (47.3 %) of the participants had resided in Zhejiang for more than 3 years. Of the 1316 participants, 73.7 % had some insurance plans, and 30.3 % had a bachelor’s or higher degree. The proportion of students among them was very low (3.5 %). The proportion of MSM with a monthly income of less than 3000 yuan (RMB) was 31.8 %. Nearly half (47.5 %) of the MSM reported their sexual orientation as homosexual or gay, and 86.7 % of the participants had had anal sex with other men in the past 6 months, out of which the proportion of participants who consistently used condoms did not exceed half. About half of the MSM had anal intercourse with at least two men in the last 6 months, and 11.3 % of the participants had suicidal inclination. It is noteworthy that 42.7 % of the respondents had a self-perceived view that they could not be infected with HIV.

### The rate of prior HIV testing, and the prevalence of HIV and syphilis

830 (63.1 %) individuals out of 1316 participants reported ever having been tested for HIV and the RDS-adjusted rate of prior HIV testing was 55.9 % (95 % CI, 51.8–60.6 %). The RDS-adjusted rates of prior HIV testing in Hangzhou, Ningbo, and Wenzhou were 62.8 % (95 % CI, 54.4–70.3 %), 61.9 % (95 % CI, 54.0–69.5 %), and 48.0 % (95 % CI, 40.9–55.0 %) respectively, while the crude rates of prior HIV testing in these cities were 67.9 %, 70.9 %, and 51.5 %, respectively. The crude rates of HIV and syphilis infections among MSM in Zhejiang Province were 14.7 % and 15.3 %, respectively, while the RDS-adjusted rates of HIV and syphilis infections among MSM in Zhejiang Province were 14.0 % (95 % CI, 11.2–17.3 %) and 11.4 % (95 % CI, 9.0–14.2 %), respectively (Table 1).

**Table 1:** Crude and RDS-weighted rates of prior HIV testing, HIV and syphilis among MSM in Zhejiang Province

Variables	Crude rate % (n/N)	RDS-weighted rate (95 % CI)
Prior HIV testing rate	63.1(830/1316)	55.9 % (51.8 %-60.6 %)
HIV prevalence	14.7(193/1316)	14.0 % (11.2 %-17.3 %)
Syphilis prevalence	15.3(201/1316)	11.4 % (9.0 %-14.2 %)

*MSM* men who have sex with men, *RDS* respondent-driven sampling, *95* % *CI* 95 % confidence interval

### Factors associated with prior HIV testing

Results of the univariate logistic regression analyses are shown in Table 2. Factors such as older age, ‘never-been-married’ marital status, higher educational levels, higher monthly income, self-reported homosexuality, suicidal inclination, self-perception of HIV infection, estimate of HIV prevalence among MSM, a higher frequency of receiving AIDS/STIs educational intervention in the last year were associated with a history of prior HIV testing.

**Table 2:** Univariate analysis of factors associated with prior HIV testing among MSM in Zhejiang Province

Variables	Total	No HIV testing n (%)	HIV testing n (%)	RDS-weighted OR (95 % CI)	*p*-value
Age(years)					
< =24	291	111(38.1)	180(61.9)	1.00	0.007
25-	555	182(32.8)	373(67.2)	1.63(1.23-2.16)	
35-	312	122(39.1)	190(60.9)	1.41(1.02-1.95)	
45-	158	71(44.9)	87(55.1)	1.23(0.83-1.81)	
Marital status					
Never married	849	294(34.6)	555(65.4)	1.00	0.050
Married/Once married	466	192(41.2)	274(58.8)	0.79(0.63-1.00)	
Education level					
Junior school or lower	547	250(45.7)	297(54.3)	1.00	<0.001
High school	369	114(30.9)	216(69.1)	1.50(1.14-1.96)	
College or higher	399	122(30.6)	277(69.4)	2.02(1.55-2.64)	
Monthly income(RMB)					
< 3000	418	184(44.0)	234(56.0)	1.00	0.001
3000-	424	144(34.0)	280(66.0)	1.57(1.19-2.06)	
4000-	474	158(33.3)	316(66.7)	1.51(1.17-1.97)	
Sexual orientation					
Homosexual	625	202(32.3)	423(67.7)	1.36(1.09-1.70)	0.007
Heterosexual or bisexual or unknown	690	283(41.0)	407(59.0)	1.00	
Had anal intercourse with men in the past 6 months					
Yes	1141	407(35.7)	734(64.3)	1.33(0.99-1.78)	0.051
No	175	79(64.6)	96(54.9)	1.00	
The number of the same sex partner whom a man had anal sex with in the past 6 months					
0	175	79(45.1)	96(54.9)	1.00	0.075
1	477	162(34.0)	31 5(66.0)	1.25(0.91-1.70)	
> =2	664	245(36.9)	419(63.1)	1.44(1.05-1.98)	
Consistent condom use during anal intercourse in the past 6 months					
Yes	564	179(31.7)	385(68.3)	1.15(0.90-1.47)	0.254
No	566	226(39.9)	340(60.1)	1.00	
Suicidal inclination					
No	1166	422(36.2)	744(63.8)	1.00	0.013
Yes	149	64(43.0)	85(57.0)	0.64(0.45-0.91)	
Self-perceived possibility of HIV infection					
Probable	48	13(27.1)	35(72.9)	1.00	0.038
Possible	225	75(33.3)	150(66.7)	0.41(0.19-0.88)	
Unlikely	477	178(37.3)	299(62.7)	0.42(0.20-0.88)	
Impossible	559	215(38.5)	344(61.5)	0.36(0.18-0.74)	
Estimate of HIV prevalence among MSM					
< =5 %	428	195(45.6)	233(54.4)	1.00	0.013
6-10 %	372	123(33.1)	249(66.9)	1.20(0.91-1.60)	
> =11 %	513	168(32.7)	345(67.3)	1.49(1.14-1.94)	
Frequency of receiving AIDS/STIs educational intervention in the past year					
Never	526	351(66.7)	175(33.3)	1.00	<0.001
Once or twice	538	93(17.3)	445(82.7)	7.50(5.71-9.85)	
More than twice	248	41(16.5)	207(83.5)	8.99(6.09-13.26)	

*MSM* men who have sex with men, *STI* sexually transmitted infection, *95* % *CI* 95 % confidence interval, *RDS* respondent-driven sampling

A weighted multivariate analysis showed that a history of prior HIV testing was associated with older age (adjusted odds ratio [AOR] = 1.91, 1.96 and 2.68 for 25–34 years old, 35–44 years old, and more than 44 years old respectively, compared to less than 25 years old); higher educational level (AOR = 1.64 and 2.10 for high school and college or higher respectively, compared to junior school or below); homosexuality (AOR = 1.31; 95 % CI, 1.00–1.73; *P* = 0.049); having suicidal inclination (AOR = 0.47; 95 % CI, 0.31–0.72; *P* = 0.001); higher frequency of receiving AIDS/STIs educational intervention in the last year (AOR = 8.27 and 11.39 for once or twice, and more than twice respectively, compared to never received intervention); and self-perceived possibility of HIV infection (AOR = 0.23, 0.28, and 0.24 for possible, unlikely and impossible, respectively, compared to probable) (Table 3).

**Table 3:** Multivariate analysis of factors associated with prior HIV testing among MSM in Zhejiang Province

Variables	AOR	95%CI	*p*-value
Age(years)			
< =24	1.00		<0.001
25-	1.91	(1.35-2.71)	
35-	1.96	(1.30-2.97)	
45-	2.68	(1.64-4.38)	
Education level			
Junior school or less	1.00		<0.001
High school	1.64	(1.18-2.29)	
College or higher	2.10	(1.46-3.03)	
Monthly income(RMB)			
< 3000	1.00		0.027
3000-	1.55	(1.12-2.15)	
4000-	1.30	(0.92-1.81)	
Sexual orientation			
Homosexual	1.31	(1.00-1.73)	0.049
Heterosexual or bisexual or unknown	1.00		
Suicidal inclination			
No	1.00		0.001
Yes	0.47	(0.31-0.72)	
Frequency of receiving AIDS/STIs educational intervention in the past year			
Never	1.00		<0.001
Once or twice	8.27	(6.19-11.07)	
More than twice	11.39	(7.50-17.29)	
Self-perceived possibility of HIV infection			
Probable	1.00		0.005
Possible	0.23	(0.10-0.54)	
Unlikely	0.28	(0.12-0.63)	
Impossible	0.24	(0.11-0.54)	

*MSM* men who have sex with men, *AOR* adjusted odds ratio, *95* % *CI* 95 % confidence interval

## Discussion

Increasing HIV testing in high-risk groups can not only help more HIV-infected people to gain knowledge of their infection status and accept treatment and care much earlier in order to prolong life and improve their quality of life, but can also effectively prevent the spread of HIV [21]. In this study, the adjusted rate of prior HIV testing in Zhejiang Province was 55.9 %, which is lower than the reported rates in Beijing (69.9 %) [22] and higher than the prior HIV testing rate in the whole country in 2009 (51.2 %) [23]. However, there is still a great disparity in HIV testing rates between Zhejiang Province and developed countries, such as Australia (80.5 %) [14] and the USA (90 %) [15].

Based on our study, higher education level was positively associated with HIV testing during a person’s lifetime, in accordance with a recent study by Zhang et al. [18]. MSM with a bachelor’s degree or higher degree were more likely to have an HIV test, probably because they perceived that they were at a higher risk for HIV infection. Our study further demonstrated that the older MSM more frequently reported as having been tested for HIV, consistent with previous reports [24–28]. Other studies also indicated that younger MSM, compared with older MSM, were less likely to undergo HIV testing [9, 29, 30]. The increase in the prevalence of HIV testing with age may be interpreted using unmeasured factors including accumulated risk, increased HIV/AIDS-related knowledge and knowledge of HIV testing, and increased exposure to social norms that promote HIV testing [31].

We also found a significant association between homosexuality and prior HIV testing. MSM with homosexual orientation tended to have an HIV test. This may be related to a lower level of internalized negative feelings about homosexuality among these MSM. Lower levels of internalized homonegativity are also associated with higher rate of undergoing STI testing [32]. Some studies have indicated that gay self-identification is associated with a history of prior HIV testing, which may be due to increased access and messages to HIV testing services and prevention, and norms of testing [33].

In this study MSM with suicidal inclination were less likely to have a test for HIV. This may be because these MSM greatly fear that they have been infected with HIV, and because they are concerned about discrimination and stigma from society, leading to the generation of their thoughts of committing suicide and no uptake of HIV testing. In the USA, MSM who are out to their health care provider as homosexual or bisexual men, are more likely to be screened for HIV [33]. However, in China, homosexuality is not accepted and is denied by the society, and MSM may face homosexuality-related and HIV-related stigma in the society [34]. This may cause negative feelings such as suicidal inclination, and may be another barrier for having an HIV test [22, 34, 35]. The mental barrier for having an HIV test is a result of the fear of being known for their homosexual behaviour, and of being suspected by the community, colleague, or boss that they were infected with HIV [34]. More interventions and public education could challenge and reduce this anti-gay prejudice and social stigma against homosexuality, which could lead to more MSM being willing to be tested for HIV. It is also critical to improve the prevalence of HIV testing by providing psychological support in HIV testing clinics, improving the general attitude of the public towards people living with HIV/AIDS, and spreading awareness about the benefits of early antiretroviral therapy.

MSM with lower self-perceived risk of HIV infection were also less likely to have an HIV test. Studies have confirmed that there is a strong association between the perception of low risk of HIV infection and a history of HIV testing [13]. A study by Zhang et al. [18] demonstrated that the perception of low risk of HIV infection and not knowing the location of the testing place are the two main factors for not taking an HIV test. We should strongly promote health education about HIV/AIDS among MSM, and improve their risk awareness of HIV infection.

Compared to MSM who did not receive intervention, those who received AIDS/STI educational intervention in the last year were more likely to have an HIV test. In the last year, we adopted AIDS/STIs educational intervention including providing condoms and lubrication, examining and treating STIs, issuing educational material, and training to improve knowledge about AIDS/STIs, which may help to increase the prevalence of HIV testing among MSM. MSM receiving AIDS/STIs educational intervention more than twice in the last year had an 11-fold (AOR = 11.39, 95 % CI, 7.50–17.29) greater likelihood of having an HIV test, compared to MSM who did not receive intervention, suggesting that the frequency of educational intervention for HIV/AIDS should be increased and expanded in our future efforts.

Our study failed to demonstrate the association between the proportion of MSM who reported ever having an HIV test and the number of male sex partners in the last 6 months. However, several studies have shown that having multiple male sexual partners in their lifetime of MSM is significantly associated with prior HIV testing [16, 17]. In our study, the number of male sexual partners during the last 6 months and the history of HIV testing during the entire lifetime of MSM was considered. We also failed to show that UAI in the last 6 months was associated with prior HIV testing, consistent with a study by Do et al. [17]. However, a study by Sifakis et al. revealed the association between recent UAI and prior HIV testing, suggesting that MSM engaging in high risk sex were more likely to undergo an HIV test [16]. These findings are contrary to the study by Zhang et al. showing that MSM who used condoms during anal sex in the last 6 months were more likely to have an HIV test, possibly because MSM who used condoms had more information regarding safe sex prior to HIV testing and counselling, leading to their condom use [18]. More extensive studies are needed to clarify the association between multiple male sex partners in the last 6 months and prior HIV testing, as well as the association between recent UAI and prior HIV testing.

The proportion of MSM who reported ever having an HIV test in Zhejiang Province is still rather low. Effective interventional measures should be adopted to improve risk awareness of HIV infection, targeting MSM population at a younger age with a low educational background. Innovative interventional measures are needed to improve the prevalence of HIV testing, such as multimedia social marketing campaigns and internet-based interventions using QQ or Wechat groups (two most popular social interaction tools used by people in China today), websites or chat rooms [36]. Social marketing campaigns are one of the effective ways of HIV prevention, which promote changes in social behaviour by culturally appropriate messages and by the use of various communication channels. They have been used to promote a large increase in HIV testing among gay and bisexual men in Toronto, Ottawa [37] and in London [38]. Rapid oral HIV testing is also an important means to improve the prevalence of HIV testing, and it may be preferred and used more frequently if conducted at clinics or at home, due to being less invasive, having easy access to testing, and may lead to less anxiety while waiting for the test results [39]. More efforts are required to encourage society to abolish discrimination and stigma against HIV-infected individuals. But perhaps a more short-term and achievable goal, particularly for promoting HIV testing, would be to reduce stigma and discrimination within health care settings, for both HIV and MSM [40, 41]. Frequent educational intervention for HIV/AIDS is needed to encourage more MSM to undergo an HIV test.

There are several limitations in our study. First, the causality of HIV testing and associated factors could not be inferred due to the cross-sectional study design. Second, the relevant information about testing-related variables is subject to recall bias, which may influence the magnitude of their associations with HIV testing, to some extent. In addition, although the RDS method is a type of approximate probability sampling for a hidden population, we have to assume that participants from the social network recruit companions randomly, to produce an estimated analytical result based on the sample characteristics; in some cases, this assumption is illegal, and the no sampling error may also present an estimated bias [42]. Lastly, the main purpose of the original survey was to obtain information regarding HIV prevalence and its associated factors; we did not entirely consider including certain measures known to be associated with HIV testing, such as social stigma. Therefore, the survey was not originally powered to detect associations with HIV testing, and true associations could be masked by the lack of power.

## Conclusions

The prevalence of prior HIV testing among MSM in Zhejiang Province, China is still rather low. Effective interventional measures should be adopted to improve risk awareness of HIV infection, targeting the younger MSM population with a lower educational background. More frequent educational intervention for HIV/AIDS is needed to abolish discrimination and stigma they face from society, provide stronger social support, and promote more MSM to take an HIV test. Innovative interventional measures are urgently needed to improve the HIV testing rate among Chinese MSM.

## Abbreviations

95 % CI: 95 % confidence intervals
AOR: Adjusted odds ratio
HIV: Human immunodeficiency virus
MSM: Men who have sex with men
RDS: Respondent-driven sampling
STI: Sexually transmitted infection
UAI: Unprotected anal intercourse
VCT: Voluntary counseling and testing
